# Racism in occupational therapy: “It’s part of who we are . . .”

**DOI:** 10.1177/03080226231153345

**Published:** 2023-02-18

**Authors:** Brenda L. Beagan, Stephanie R. Bizzeth, Tara M. Pride, Kaitlin R. Sibbald

**Affiliations:** School of Occupational Therapy, Dalhousie University, Halifax, NS, Canada

Since the resurgence of the Black Lives Matter movement in 2020, statements of positionality in relation to racism, territorial acknowledgements, and proclamations against systemic racism have become more common. This is a good thing. Positioning in relation to racism matters. Yet such statements too often lack social, historical, and political context, lack analysis of root causes of racism, and rarely move toward actions. They risk becoming mere performances. In this editorial, we contextualize the positions from which we write about racism, in Eastern Canada, to show the ways colonial racism is inseparable from the construction of nations, cultures, and institutions. Drawing on the anti-racism commitments of numerous occupational therapy organizations, we examine evidence for and impacts of racism within occupational therapy, and we generate ideas for action.

## Positioning ourselves in a nation built on colonial racism

We write today from Mi’kma’ki, territory the Mi’kmaq have lived in and cared for since time immemorial. The First Peoples of this place, never “gave it up,” never “lost” it to the victors as the spoils of war. Rather they signed Treaties of Peace and Friendship with the British Crown in 1725, 1729, 1749, 1779, with both sides agreeing to live in harmony, respecting the rights of the other. Also in 1749 (when not signing friendship treaties) Governor Edward Cornwallis, representing the British Crown, signed a declaration offering a £25 bounty for any Mi’kmaw scalp, an even higher price for Mi’kmaq brought in alive (women and children were worth a bit less). That decree was rescinded, then reissued in 1756 by Governor Charles Lawrence, and has never been repealed (Paul, 2006; Tattrie, 2013). One of us is a Mi’kmaw woman. Presumably, the rest of us could turn her in, somewhere, and claim the bounty.

This forested land was gradually cleared, while the British and French fought for sovereignty. During their first 150 years in Mi’kma’ki, the French-heritage Acadians formed alliances with Mi’kmaw friends and neighbors. When they refused to sign loyalty to the British Crown, they were expelled, mostly to the United States during the Expulsion of the Acadians 1755–1764. The lands they had cleared and farmed were given to British Loyalists (Marsh, 2015). The land here does not lend itself to plantation-style production, which meant involvement in the trans-Atlantic African slave trade was less prominent than it was in building the economy of the United States. But there were African slaves here, mostly as domestics, as early as the 1600s. In the 1760s as Britain defeated France for control of what is now Eastern Canada, treaties enshrined people’s rights to keep their slaves (Henry, 2022).

After the American Revolution, in the 1780s and 1790s, United Empire Loyalists leaving the United States (US) for Mi’kma’ki/Eastern Canada were expressly encouraged to bring their furniture, other goods and slaves. At the same time, land grants were offered to Black United Empire Loyalists, former slaves and freedmen from the US who had fought for Britain. For the approximately 3500 who came to what is now Nova Scotia, promises of 100 acres per household materialized as about ¼ acre each. They lived in small Black communities, impoverished and facing constant hostility and violence (Bonikowsky, 2019). One of us is seventh generation African Nova Scotian, with roots in Nova Scotia stretching back to the early-1800s arrival of an ancestor from Bermuda, another former British colony.

As Britain fought to establish colonial control in Canada’s East coast, settlers were enticed to come here, clear land, build properties, and farm. Some were given huge land grants, then recruited tenants to clear and settle on that land. Many tenants were not well-off, having been subject to English occupation and “clearances” in Ireland and Scotland, and they worked hard at great sacrifice to establish themselves in their new country. But most were given land, arable land, unlike the Black Loyalists. That land settlers were “given” was stolen from the Mi’kmaq. Meanwhile, Indigenous Peoples were pushed into smaller and smaller territories, eventually (under the Indian Act 1876) a system of “reserves,” land tracts not owned by, but occupied by Indigenous communities. These lands were isolated, often barely arable, allowing settlers unimpeded access to the rich resources around them. Eventually, Indigenous Peoples needed a pass to leave the reserve lands, and needed permits to sell their produce off-reserve. One of us descends from British United Empire Loyalists, and from Northern Scots who arrived in 1773, promised free passage, 1 year of free provisions, and a farm. The “farm” was uncleared forest, the provisions not forthcoming, but the Mi’kmaq helped the Scottish settlers survive (MacKay, 2001). Another of us descends mainly from Irish settlers who were given land to farm in Mi’kma’ki in the late 1700s and early 1800s; they had freedom of movement and the right to vote (at least the men did).

Why this focus on (re)writing/righting history? In response to the ubiquitous question constantly leveled at Indigenous Peoples, “Why can’t you just get over this?” Indigenous leader Senator Murray Sinclair flipped the script, asking, “Why can’t you always remember this? . . . We should never forget . . . it’s part of who we are as a nation” (CBC, The Current, 2017). Racism, inextricably reliant upon colonial capitalism, is not an aberrant act committed by isolated individuals who embrace objectionable ideologies (see Choonara, 2021). It is woven into our histories of nation-building, in Britain and its former colonies. It is written into the very fabric of our nations, written in blood and sweat and tears. Written with complacency, entitlement, righteousness, and ignorance. It was accomplished through the theft of Indigenous land and resources, ongoing domination, and attempts at cultural genocide. It was accomplished through the impoverishment and slavery of Black settlers, the exploitation of Chinese migrants to build railways while refusing to let them bring their families, the internment of Japanese Canadians during World War II while appropriating their property and possessions (Canadian Heritage, 2022). Many white Canadians do not know the histories sketched above—do not feel a need to know them, do not want to know them. Those histories disrupt well-established myths of nation-building that celebrate adventurers with pioneering spirits arriving and working hard here to succeed, thrive, and forge a nation. Cultivating determined ignorance allows the descendants of white settlers (and newer settlers) to deny the colonial racism that has been foundational to white supremacy, allowing it to thrive across centuries.

Yet racist colonial capitalism continues to rely on the extraction of resources from unceded Indigenous territories. The “standard of living” enjoyed by many Canadians is paid for by the poverty and poorer health of Indigenous Peoples who are still denied sovereignty over their territories, still denied self-governance. Resources are still actively taken from Indigenous communities and distributed to settler communities. Despite the deep-rooted colonial systems intended to eradicate them, Indigenous Peoples and communities continue to actively challenge and resist oppression. While Black people may no longer be enslaved, they face a “prison industrial complex” and a “school to prison pipeline” which leaves them working virtually for free in prisons and jails (Alexander, 2012; Morris, 2020). When Black youth make up 12% of school enrolees, but 48% of those expelled from schools, they are launched into precarious economic engagements that may lead to violence and/or incarceration (Shen, 2021). Black job applicants and those with accents or names that “sound Black” are significantly less likely to get job interviews than white and “white-sounding” applicants, using the same résumé (Branker, 2017; Henry & Ginzberg, 1985). This pattern is remarkably consistent across time and place (Hussein, 2022; Quillian et al., 2017). Black women are more likely than other Canadians to attend post-secondary education, yet have the lowest employment rates and incomes (Statistics Canada, 2020). Structures of racism grounded in colonialism and capitalism twist and distort every social institution—from politics to the media, from education to employment, from the legal system to health care—privileging white Canadians at the expense of Indigenous Peoples and racialized Canadians.

## Professional commitments to anti-racism

When dramatic events happen that bring renewed attention to racism, people rightly respond with grief, anger and condemnation. In 2020, the murder of African American George Floyd by police spurred an international resurgence of Black Lives Matter, a movement begun in 2013 in the US. Despite similar killings by police of Black people before and since George Floyd’s death, after a surge of support in 2020, endorsement of Black Lives Matter in the US has diminished (Civiqs, 2022; Rahman, 2022). Black Lives Matter also saw a 2020 resurgence in Canada, heightened by the police-involved deaths of Black and Indigenous people, D’Andre Campbell and Regis Korchinski-Paquet in April and May 2020, Chantel Moore and Rodney Levi in June 2020. In the United Kingdom (UK) and globally, protests led to the defacing and toppling of statues and monuments commemorating colonial rulers and supporters of the enslavement of African peoples. 2020 also saw the release of statements condemning racism, and committing to anti-racist actions, by several occupational therapy organizations and journals (AOTA, 2021; CAOT, 2020; Gustafsson et al., 2022; Justice-based Occupational Therapy [JBOT], 2020; RCOT, 2020a; Stanley et al., 2020; World Federation of Occupational Therapists [WFOT], 2020). Such professions of commitment to anti-racism are important, if institutions can be held accountable to them. And if the commitments are enduring. In late 2022, the discovery in Western Canada of multiple Indigenous women killed by one man was met with virtual silence (Andrews, 2022).

The earliest research on racism within occupational therapy was published in this journal (Bogg et al., 2006). In a survey of occupational therapists in the NHS (*n* = 396), only 3% identified as Black or “minority ethnic,” but half of those thought their race/ethnicity had posed a barrier to career progress. Of the entire sample, close to ¾ thought Black and minority ethnic groups were poorly represented in the profession, particularly in senior administrative positions. Fifteen years later, Black and ethnic minorities continue to report feelings of not-belonging in the profession, concerns about career progression, and awareness that the profession is grounded in white-centric worldviews (Atwal et al, 2021). In Canada, “visible minorities” make up about 15% of all occupational therapists, on par with the population overall; only 1% of occupational therapists are Black, and just over 1% are Indigenous (compared with 3.5 and 9.4% of the population) (Statistics Canada, 2019a, 2019b, 2022a, 2022b). In the UK, 10–12% of occupational therapists identify as Black, Asian or “other non-white” (Health Care and Professions Council [HCPC], 2021), compared to 18% of the population (Race Disparity Unit, 2021). Racism structures every level of the health-care system, from who is admitted for training, and how internationally-trained health professionals are credentialled (or not), to the content and processes of health professional education, to hiring practices and career progress, to the processes and technologies used in working with patients/clients (see Choonara, 2021, for an excellent structural analysis). On *top* of such structural aspects, racism shapes the beliefs and perceptions of individual health professionals in ways that impede quality care for racialized patients/clients (McConnell, 2022).

Our own research has shown that racism is part of everyday work for racialized occupational therapists (Beagan et al., 2022a, 2022b). They may feel isolated and alone, both invisible and hypervisible. They may be socially excluded by colleagues and not supported when clients are hostile and/or refuse to work with them. They may face microaggressions, tokenism, and exoticism (from colleagues and clients), while they are also dismissed as less competent. They are cast as not-credible knowers; their expertise and authority are undermined on all sides, challenging their professional status. Not surprisingly, then, racialized occupational therapists are underrepresented in power positions within the profession. All of these research results are echoed in both the US and the UK (e.g., Atwal et al., 2021; Ford et al., 2021; Hussein, 2022). Potential responses to systemic racism within the profession that are available to individual therapists all carry negative consequences (Beagan et al., 2022a): Withdraw and disengage? Assimilate to cultures of whiteness as much as possible, denying oneself? Resist, speak out, and be cast as “a trouble-maker?” Shoulder responsibility, trying to do things “just right” to avoid the harms of racism? Choose your battles, resisting sometimes and letting it go sometimes? Laugh it off (again)? Invest extraordinary time/energy/emotion pursuing redress under anti-discrimination policies that rarely work in the long run? One of the few responses that appears to hold promise is finding community with others who have similar experiences and/or similar analyses of equity and justice issues.

## Making change

The Royal College of Occupational Therapists has stated regarding anti-Black racism, “There is a lot of work to be done to eradicate inequalities, and we are committed to playing our part” (RCOT, 2020b). How then do we bring about change? Systemic racism operates at multiple, interconnected levels. They get labelled differently by different scholars, but in effect encompass interpersonal racism (between individuals), institutional racism (built into the policies, procedures, and practices of institutions), and structural racism (broad societal level patterns of inequity, including ideologies, belief systems, and access to resources). At the interpersonal level, change requires those of us who benefit from racism to take responsibility for white supremacy: learning about everyday racism and microaggressions; developing awareness of when we discount the knowledges of racialized colleagues and clients; acknowledging our racist actions and inactions, words, and silences; developing the skills to speak up when we encounter racism; amplifying the voices and actions of racialized coworkers; listening.

Confronting racism within occupational therapy also means taking steps to transform institutions, using whatever power and privilege each of us has to do so. This means developing structural competency, learning to think critically to understand the roots of white supremacy in the profession and in our institutions. Critical analysis can be learned—*should* be learned (Nixon et al., 2017). We can learn to ask the hard questions, like whose worldviews are embedded in our assessments and conceptual models, whose knowledges and ways of knowing are valued and taken up, who is seen as a helper and who as in need of help, who is seen as authoritative and embodying leadership? When examined critically, even core concepts like client-centeredness and enablement betray their roots in privileged, dominant worldviews and experiences (Beagan et al., 2022c; Emery-Whittington, 2021; Gibson, 2020; Grenier, 2020; Hunter & Pride, 2021). We can use institutional mechanisms to transform curricula, support racialized colleagues, and advance racialized colleagues into positions of power and leadership. We can insist on making space for the voices and writings of racialized therapists and scholars (Johnson & Lavalley, 2021). We can transform recruitment, admissions, hiring, and promotion practices to make space for non-normative ways of knowing, doing, and being. We can help to set up (and fund) processes for peer support, mentorship and communities of practice (if they do not exist locally) for racialized students and therapists (Ford et al., 2021; Hussein, 2022). At a more everyday level, we can quietly practice resistance, becoming “disobedient” occupational therapists (Turcotte & Holmes, 2021), such as implementing anti-oppressive documentation (MacLachlan & Grenier, 2022).

At a structural level, we can learn to recognize the intersections of racism, colonialism and capitalism; analysis then demands action. Proclamations of commitment must be accompanied by deepening and broadening the struggle, work that goes beyond critical interrogation and reflexivity at the individual or even institutional level (Choonara, 2021). At a time of deep divisions in Western societies, it demands strategic allegiances and coalitions, collective rather than individual action. It requires engagement with communities and existing activist groups, with unions and nonprofits, with faith-based groups and politicians. It means finding bases of unity with groups fighting for other causes.

If this sounds challenging—it is. Luckily within occupational therapy we already have leaders showing us the way. In Brazil, social occupational therapy has been confronting individualism and capitalism for decades, such as the work of Esquerdo Lopes and Serrata Malfitano (2021). In Australia, New Zealand, and Canada, Indigenous therapists and scholars are leading the way in decolonizing practice, such as the work of Chontel Gibson (2020), Isla Emery-Whittington (2021), Angie Phenix, Kaarina Valavaara (e.g., Restall et al., 2019), and Tara Pride (e.g., Hunter & Pride, 2021). In South Africa, occupational therapists like Roshan Galvaan and Elelwani Ramugondo are directing us to new ways of thinking and doing (e.g. Galvaan & Rauch van der Merwe, 2021; Ramugondo, 2018). And in America, Khalilah Johnson and Ryan Lavalley have been pushing for anti-racist praxis in occupational therapy (e.g. Johnson et al., 2022). Are we prepared to go beyond protestations of outrage (Jean-Pierre & James, 2020), and promises to “promote diversity and inclusion” (RCOT, 2020a)? Are we prepared to engage in action, upholding occupational rights for all?
